# Dual Identification and Analysis of Differentially Expressed Transcripts of Porcine PK-15 Cells and *Toxoplasma gondii* during *in vitro* Infection

**DOI:** 10.3389/fmicb.2016.00721

**Published:** 2016-05-13

**Authors:** Chun-Xue Zhou, Hany M. Elsheikha, Dong-Hui Zhou, Qing Liu, Xing-Quan Zhu, Xun Suo

**Affiliations:** ^1^National Animal Protozoa Laboratory and College of Veterinary Medicine, China Agricultural UniversityBeijing, China; ^2^State Key Laboratory of Veterinary Etiological Biology, Key Laboratory of Veterinary Parasitology of Gansu Province, Lanzhou Veterinary Research Institute, Chinese Academy of Agricultural SciencesLanzhou, China; ^3^Faculty of Medicine and Health Sciences, School of Veterinary Medicine and Science, University of NottinghamLoughborough, UK; ^4^Jiangsu Co-innovation Center for the Prevention and Control of Important Animal Infectious Diseases and Zoonoses, Yangzhou University College of Veterinary MedicineYangzhou, China

**Keywords:** *Toxoplasma gondii*, PK-15 cells, host-pathogen interaction, transcriptome, KEGG, protein-protein interaction

## Abstract

*Toxoplasma gondii* is responsible for causing toxoplasmosis, one of the most prevalent zoonotic parasitoses worldwide. The mechanisms that mediate *T. gondii* infection of pigs (the most common source of human infection) and renal tissues are still unknown. To identify the critical alterations that take place in the transcriptome of both porcine kidney (PK-15) cells and *T. gondii* following infection, infected cell samples were collected at 1, 3, 6, 9, 12, 18, and 24 h post infection and RNA-Seq data were acquired using Illumina Deep Sequencing. Differential Expression of Genes (DEGs) analysis was performed to study the concomitant gene-specific temporal patterns of induction of mRNA expression of PK-15 cells and *T. gondii*. High sequence coverage enabled us to thoroughly characterize *T. gondii* transcriptome and identify the activated molecular pathways in host cells. More than 6G clean bases/sample, including >40 million clean reads were obtained. These were aligned to the reference genome of *T. gondii* and wild boar (*Sus scrofa*). DEGs involved in metabolic activities of *T. gondii* showed time-dependent down-regulation. However, DEGs involved in immune or disease related pathways of PK-15 cells peaked at 6 h PI, and were highly enriched as evidenced by KEGG analysis. Protein-protein interaction analysis revealed that *TGME49_120110* (*PCNA*), *TGME49_049180* (*DHFR-TS*), *TGME49_055320*, and *TGME49_002300* (*ITPase*) are the four hub genes with most interactions with *T. gondii* at the onset of infection. These results reveal altered profiles of gene expressed by PK-15 cells and *T. gondii* during infection and provide the groundwork for future virulence studies to uncover the mechanisms of *T. gondii* interaction with porcine renal tissue by functional analysis of these DEGs.

## Introduction

Infection with the apicomplexan protozoan parasite *Toxoplasma gondii* can cause severe disease and even death in immune-compromised individuals and in congenital infections (Elsheikha, 2008). However, association between parasite genotype, host genetic background and severity of the disease may occur in healthy subjects (Bela et al., 2012; McLeod et al., 2012; Xiao and Yolken, 2015). *T. gondii* strains from Europe and North America have been reported to belong to three (type I, II, and III) main evolutionary lineages (Howe and Sibley, 1995; Khan et al., 2011; McLeod et al., 2012). Genotypes not belonging to the three lineages have been detected in South America (Pena et al., 2008). Recent studies revealed even more genetic diversities, which seem to be driven via genetic recombination events that occur during the sexual phase of the life cycle in the gut epithelium of the definitive felid host (Minot et al., 2012).

What makes *T. gondii* so special compared to other apicomplexan protozoa is its ability to infect any nucleated cell types in virtually all warm-blooded animals (Dubey et al., 1998; Dubremetz, 1998; Schlüter et al., 2014; Yarovinsky, 2014). Successful infection of *T. gondii* tachyzoites depends on their ability to contend with immune responses mounted by the hosts they infect. *T. gondii* organisms have a remarkable ability to manipulate host cell biological process to their own advantage and to evade both innate and adaptive host immune defenses (Hunter and Sibley, 2012; Coombes and Hunter, 2015). Hence, if *T. gondii* tachyzoites are to replicate intracellularly and survive long enough to effectively establish infection they need to exploit host processes that are beneficial to their metabolic, anti-apoptotic and pathogenetic functions. The host cells, on the other hand, employ several strategies, such as repair and stress pathway, to adapt to and mitigate the damage caused by the parasite (Gazzinelli et al., 2014).

Interaction between *T. gondii* and host cells is complex due to the numerous parasite and host factors mediating this interaction. *T. gondii* invades host cells and establishes a parasitiphorous vacuole (PV) in the host cell cytoplasm in which it resides (Pissuwan et al., 2007). Within the PV, *T. gondii* undergoes endodyogeny, assembling two daughter parasitic cells within each parental parasite in every mitotic cell cycle (~7–10 h post infection; Hu et al., 2002). The main events of *T. gondii* cell cycle, G1, single S and mitotic (S/M) phases, and cytokinesis (C) phase (Butler et al., 2014), are associated with a coordinated program of gene expression as revealed by microarray and RNA-Sequencing analyses (Behnke et al., 2010; Gaji et al., 2011). During this intracellular replication cycle, specific host cellular state can influence *T. gondii* gene expression (Radke et al., 2006) and *T. gondii* secreted effectors could modulate host gene expression leading to an altered host cell microenvironment to which it subsequently responds. The correlations between host and *T. gondii* gene expression clusters (Melo et al., 2013) clearly support the presence of coordinated cross-talks that mediate interaction between *T. gondii* and host cells.

The remarkable capability of *T. gondii* to exploit surrogate host cells have spurred extensive research on the relationship between this parasite and its host using various technologies, including proteomics (Nelson et al., 2008; Zhou et al., 2011, 2013), metabolomics (Tymoshenko et al., 2015) and transcriptomics (Melo et al., 2013; He et al., 2016). Specifically, high-throughput RNA-Sequencing can provide a powerful method for identifying novel RNAs and for studying the expression profiles of genes in different biological samples (Melo et al., 2013), such that hundreds of differentially expressed genes (DEGs) can be identified and quantified in a single analysis. The high sensitivity of RNA-seq can facilitate the precise identification of the DEGs within particular cells or tissues (Sansom et al., 2014).

The pathogenesis of *T. gondii* in various body tissues has been extensively studied, but little is known about how *T. gondii* interacts with host renal tissue. Also, although pig is a natural host of *T. gondii* and represents a major source of zoonotic infection (Tenter et al., 2000; Steinparzer et al., 2015), knowledge of the pathophysiology of toxoplasmosis in this intermediate host species has been very limited. Further, little is known about DEGs and the role they play during *T. gondii* infection. To address these needs, the present study was performed to investigate the concurrent transcriptomic changes of *T. gondii* and porcine kidney (PK-15) host cells spanning the first 24 h of infection using next-generation mRNA sequencing (RNA-seq). Quantitative real-time PCR was also performed to validate the transcriptome sequencing data. The obtained transcriptomic sequences were mapped to reference genomes, and were analyzed to identify unique regulatory pathways that are associated with *T. gondii* during the first 24 h of infection. Understanding of these pathways may provide information likely to be critical for the development of rationally designed *T. gondii* therapeutics and vaccines.

## Materials and methods

### Cell culture

Porcine kidney (PK-15) cells were purchased from National Platform of Experimental Cell Resources for Sci-Tech (Beijing, China) and cultured in Dulbecco's modified Eagle medium (DMEM, HyClone, China) supplemented with 10% heat-inactivated fetal bovine serum (FBS, Gibco, USA), 100 units/mL penicillin and 100 μg/mL streptomycin (Invitrogen), and incubated at 37°C in a humidified 5% CO_2_ atmosphere.

### *In vitro* parasite cultivation and infection of PK-15 cells

Type I virulent *T. gondii* cultures of RH strain tachyzoites were maintained in our lab in immortalized PK-15 epithelial cells. Briefly, monolayers of PK-15 cells, at 80% confluence in 25-cm^2^ culture flasks, were infected with *T. gondii* tachyzoites at a multiplicity of infection (MOI) of 5. Tachyzoites were harvested from host feeder cells when 70–80% of infected PK-15 epithelial cells had lyzed. The egressed tachyzoites and the remaining infected cells were harvested with a cell scraper, passed through a syringe and 22-gauge needle to rupture remaining intact host cells, washed once in cold sterile phosphate buffered saline (PBS), and centrifuged twice at 5000 × g for 10 min to eliminate cell debris. The obtained parasite pellet was washed twice with cold PBS with centrifugation at 350 × g for 10 min (Qu et al., 2013). The final purified tachyzoites were re-suspended in PBS. The enriched parasite preparation was counted using a hemocytometer and the concentration of tachyzoites was adjusted by adding fresh medium to achieve a desired MOI of 5, i.e., five tachyzoites per cell. Infected PK-15 cells were incubated for 1 h, followed by removal of the culture medium, washing three times with PBS to remove unattached tachyzoites and addition of fresh culture medium. Infected cultures were incubated and at 1, 3, 6, 9, 12, 18, or 24 h PI samples from infected culture and controls were collected and processed for sequencing analysis. Mock-infected cells and purified parasites were used as controls. Infection experiment with *T. gondii* was performed under biosafety level 2 (BSL-2) conditions. All infections were performed at 37°C in an atmosphere of 5% CO_2_.

### RNA isolation, cDNA library preparation and illumina deep sequencing

RNA was extracted from samples at various times post infection by using the PrimeScript™ 1st Strand cDNA Synthesis Kit (Takara Bio, Shiga, Japan) according to the manufacturer's instructions. Total RNA was treated with PQI DNase (Promega, MI, USA) to eliminate the residual DNA contamination. RNA degradation and contamination were monitored on 1% agarose gels. The purity was checked using the NanoPhotometer^®^ spectrophotometer (IMPLEN, CA, USA) and the integrity was assessed using the RNA Nano 6000 Assay Kit of the Bioanalyzer 2100 system (Agilent Technologies, CA, USA). Finally RNA concentration was measured using Qubit^®^ RNA Assay Kit in Qubit^®^ 2.0 Flurometer (Life Technologies, CA, USA).

The cDNA synthesis was performed with 3 μg of total RNA using NEBNext^®^ Ultra™ RNA Library Prep Kit for Illumina^®^ (NEB, USA) following the manufacturer's recommendations. Briefly, mRNA was selected by using poly-T oligo-attached magnetic beads and then fragmented using divalent cations under elevated temperature in NEBNext First Strand Synthesis Reaction Buffer (5X). Reverse transcription was performed using random hexamer primer and M-MuLV Reverse Transcriptase with the mRNA fragments as templates. Double stranded cDNA fragments were obtained using DNA Polymerase I and RNase H. Remaining overhangs were blunt-ended and the cDNA fragments were ploy-adenylated. NEBNext Adaptor with hairpin loop structure was ligated to prepare for hybridization. The library fragments were purified with AMPure XP system (Beckman Coulter, Beverly, USA) to obtain cDNA fragments of preferentially 150~200 bp in length. Purified DNA fragments were then enriched using Phusion High-Fidelity DNA polymerase, Universal PCR primers and Index (X) Primer in a 15-cycle PCR reaction. PCR products were purified (AMPure XP system) and library quality was assessed on the Agilent Bioanalyzer 2100 system.

Before sequencing, the clustering of the index-coded samples was performed on a cBot Cluster Generation System using TruSeq PE Cluster Kit v3-cBot-HS (Illumina) according to the manufacturer's instructions. Sequencing runs were performed on an Illumina Hiseq platform and 125 bp/150 bp paired-end reads were generated.

### Reads mapping

Raw sequencing reads (raw data) were processed through in-house perl scripts and the read counts were extracted. The raw reads were then filtered to remove low-quality sequences (Q < 20), empty reads, and reads containing adapter or poly-N to gain clean reads. All subsequent analyses were based on the clean reads with high quality. Reference genomes and gene model annotation files were downloaded (ftp://ftp.ensembl.org/pub/release-76/fasta/sus_scrofa/dna/Sus_scrofa.Sscrofa10.2.dna.toplevel.fa.gz;ftp://ftp.ensemblgenomes.org/pub/release-23/protists//fasta/toxoplasma_gondii/dna/Toxoplasma_gondii.ToxoDB-7.1.23.dna.toplevel.fa.gz). Index of the reference genome was built using Bowtie v2.2.3 and paired-end clean reads were aligned to the reference genome sequence using TopHat v2.0.12 (Zhang et al., 2014). Picard-tools v1.96 and samtools v0.1.18 were used to sort mark duplicated reads and reorder the bam alignment results of each sample (Liu et al., 2015a). GATK2 (v3.2) software was used to perform SNP calling (DePristo et al., 2011). The Cufflinks v2.1.1 Reference Annotation Based Transcript (RABT) assembly method was used to construct and identify both known and novel transcripts from TopHat alignment results. Alternative splicing (AS) events were classified to 12 basic types by the software Asprofile v1.0 (Florea et al., 2013). The number of AS events in each sample was estimated separately. To annotate all unigenes obtained, we searched them against the protein databases NR, Swiss-Prot, KEGG, COG and Toxo DB using blastx (BLAST, the basic local alignment search tool; Liu et al., 2015b).

### Differential gene expression analysis

To detect differentially expressed genes (DEGs), the number of clean reads assigned to a gene was counted using HTSeq v0.6.1. The read counts were then normalized into the values of fragments per kilobase of exon per million fragments mapped (FPKM) (Trapnell et al., 2010). DEGSeq R package (1.20.0) was utilized to perform differential expression analysis of two conditions (Robinson et al., 2010). Corrected *p*-value (*q*-value) < 0.005 and |log_2_ (fold change)| > 1 were set as the threshold for significantly DEGs. Subsequently, the enrichment analysis of Gene Ontology (GO) and KEGG pathways was performed based on these DEGs by using GOseq R package and KOBAS 2.0 software (http://kobas.cbi.pku.edu.cn/home.do) (Xie et al., 2011). Finally, predicted Protein-Protein Interactions (PPI) analysis was performed based on the STRING database (Szklarczyk et al., 2015).

### Verification by quantitative real time RT-PCR

Total RNA was extracted using TRIzol method (Invitrogen) and then was reverse-transcripted to single strand cDNA using GoScript™ Reverse Transcription System (Promega, MI, USA) according to the manufacturer's instructions. GoTaq^®^ qPCR Master Mix (Promega, MI, USA) was used according to the manufacturer's protocols to perform RT-PCR reactions on QIAGEN's real-time PCR cycler, the Rotor-Gene Q. The amplification reactions were performed with the following conditions: 2 min at 95°C, 40 cycles of 95°C for 15 s, 55°C for 30 s, 72°C for 30 s. All quantitative measurements were carried out in triplicate and normalized to the internal *glyceraldehyde-3-phosphatedehydrogenase* (*GAPDH*) control for every reaction (Schmittgen and Livak, 2008). Results were expressed as mean value ± standard deviation (SD). More than 20 significantly differentially expressed genes were selected to validate the sequencing data and sequences of forward and reverse primers used in this study are listed in Table S1. The mRNA fold change was calculated by the following equations: ^Δ^*C*_*T*_=^Δ^*C*_*T*__(target)_−^Δ^*C*_*T*__(GAPDH)_; ^ΔΔ^*C*_*T*_ = ^Δ^*C*_*T*__(infected)_−^Δ^*C*_*T*__(control)_; mRNA fold change = 2^−ΔΔ*C*_*T*_^ (Xu et al., 2012).

## Results

### Illumina sequencing and read mapping

We used RNA-sequencing (RNA-seq) of poly-adenylated RNA to capture the dynamic temporal changes of PK-15 cells and *T. gondii* RH strain transcriptomes at 1, 3, 6, 9, 12, 18, or 24 h PI. Nine sequencing libraries were prepared and sequenced with the Illumina paired-end method. More than 4.3 × 10^7^ raw reads were generated in each group (Table 1). After removing low quality reads, clean reads were obtained and >95% of the clean reads had Phred-like quality scores at the Q20 level (an error probability of 0.01) and the GC-contents were about 50%.

**Table 1:** **Quality of sequencing**.

**Sample ID**	**Raw reads**	**Clean reads**	**Clean bases**	**Error rate (%)**	**Q20^a^ (%)**	**Q30^b^ (%)**	**GC content (%)**
T0_C	43446736	41711988	6.26G	0.02	95.79	90.18	50.54
T0_T	50923560	48608268	7.29G	0.02	95.57	89.7	52.27
T1	50649082	48561200	7.28G	0.02	95.92	90.44	51.17
T3	46208992	44198772	6.63G	0.02	95.74	90.05	51.68
T6	53880410	51532412	7.73G	0.02	95.24	89.02	51.52
T9	50788268	48390640	7.26G	0.02	95.68	89.9	51.05
T12	58462716	56057010	8.41G	0.02	95.9	90.38	51.33
T18	44173198	42506698	6.38G	0.02	95.98	90.52	51.54
T24	52617190	50101660	7.52G	0.02	95.57	89.67	52.13

a *percentage is the proportion of nucleotides with a quality value >20 in reads*.

b *percentage is the proportion of nucleotides with a quality value >30 in reads*.

We then mapped the sequenced clean reads to *T. gondii* and wild boar (*Sus scrofa*) genome, respectively. The majority of the clean reads were distributed in the exon region, followed by intergenic region and the intron region. Clean reads that were not mapped to the *Sus scrofa* genome were mapped to the genome sequence of *T. gondii*. About 90% of the total clean reads that were mapped to the *Sus scrofa* genome were unique. However, above 99% of the total clean reads were uniquely mapped to the *T. gondii* genome, while less than 1% of the clean reads were mapped to both reference genomes. As shown in Figure S1, the increased total reads and the unique mapped reads to *T. gondii* genome was proportional to time after infection. In contrast, total and uniquely mapped reads to the *Sus scrofa* genome decreased as time after infection increases. All subsequent analyses were based on the uniquely mapped reads.

### Discovery and characterization of SNPs and InDels in transcriptomes

**Figure 1:**
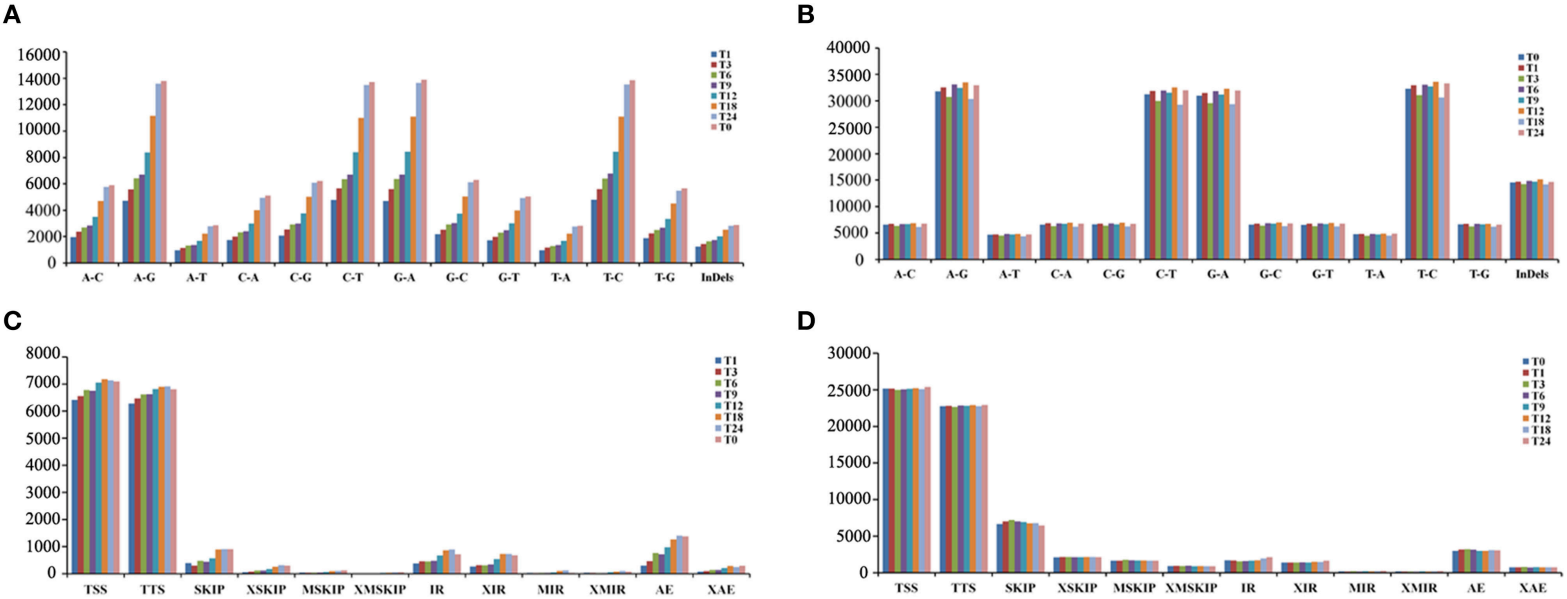
**Statistics of single nucleotide polymorphisms (SNPs) and alternative splicing (AS) events identified from the RNA-Seq data**. **(A)** Classification of putative SNPs and INDELs identified from the transcriptomes of *T. gondii*. The x-axis represents types of SNP events. The y-axis represents number of SNP events. **(B)** Classification of putative SNPs and INDELs identified from the transcriptomes of PK-15 cells. The x-axis represents types of SNP events. The y-axis represents number of SNP events. **(C)** Numbers of the AS events in *T. gondii*. The x-axis represents types of AS events. The y-axis represents number of AS events. **(D)** Numbers of AS events in PK-15 cells. The x-axis represents types of AS events. The y-axis represents number of AS events. The AS events include transcription start site (TSS), transcription terminal site (TTS), skipped exon (SKIP_ON,SKIP_OFF pair) (SKIP), approximate SKIP (XSKIP_ON,XSKIP_OFF pair) (XSKIP), multi-exon SKIP (MSKIP_ON,MSKIP_OFF pair) (MSKIP), approximate MSKIP (XMSKIP_ON,XMSKIP_OFF pair) (XMSKIP), intron retention (IR_ON, IR_OFF pair) (IR), approximate IR (XIR_ON, XIR_OFF pair) (XIR), multi-IR (MIR_ON, MIR_OFF pair) (MIR), approximate MIR (XMIR_ON, XMIR_OFF pair) (XMIR), alternative exon ends (5′, 3′, or both) (AE), and approximate AE(XAE).

RNA-Seq is an efficient way to comprehensively identify single nucleotide polymorphisms (SNPs) and Insertions/Deletions (InDels) events from the expressed genes. Putative SNPs were predicted from *T. gondii* infected and control samples based on read depth and quality score of alignment results. Among the detected SNPs, A/G, G/A, C/T, and T/C transitions, were the most abundant and equally distributed (Figures 1A,B). For SNPs transversion, A/T and T/A were the smallest types. Alternative splicing (AS) is an important regulatory mechanism underlying increased cellular and functional complexity in eukaryotes and plays an important role in the generation of proteomic and biological diversities (Pan et al., 2008). Among the 12 basic types of AS events, transcription start site (TSS) and transcription terminal site (TTS) were the two major events (Figures 1C,D). Additionally, the number of putative SNPs and InDels identified from *T. gondii* transcriptomes increased proportional to time after infection. The AS events showed a similar trend.

### Dynamics of gene expression

**Figure 2:**
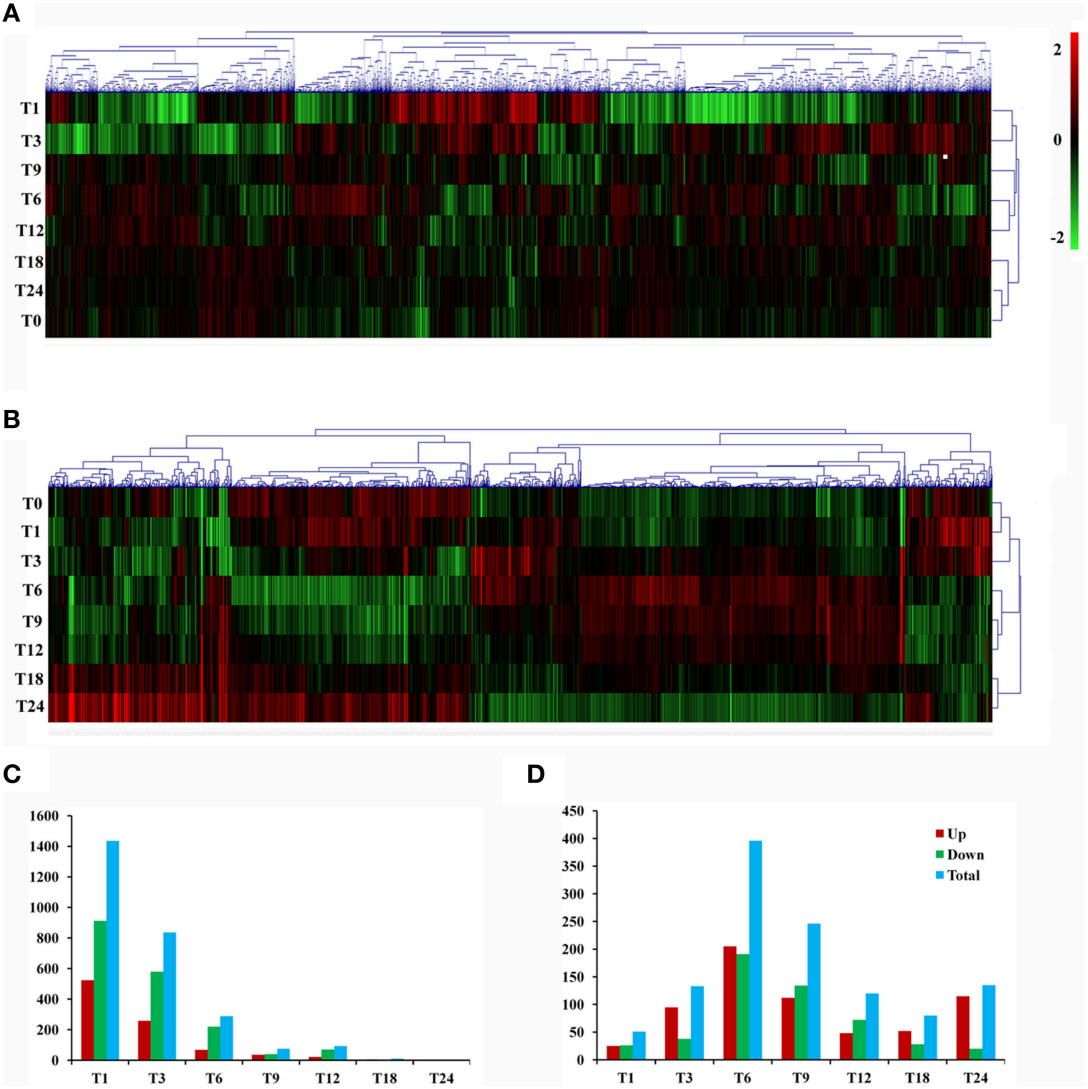
**Gene expression profiles during *T. gondii* infection**. **(A)** Hierarchical clustering of differentially expressed genes of *T. gondii* during infection. Expression values (FPKM) were log_2_-transformed and subsequently median-centered by gene. Rows were hierarchically clustered based on average linkage using Pearson correlation coefficients as the distance measure. The expression levels are visualized using gradient color scheme, the scale from least abundant to highest range is from -2.0 to 2.0. Green color indicates low expression, and red color indicates high expression of the detected genes. The left vertical axis represents sample ID. The horizontal axis shows clusters of samples and the above vertical axis shows clusters of DEGs. **(B)** Hierarchical clustering of differentially expressed genes of *T. gondii*-infected PK-15 cells. **(C)** Numbers of DEGs in the parasites during infection process. **(D)** Numbers of DEGs in the host PK-15 cells during infection process. Up, up-regulated DEGs; Down, down-regulated DEGs; Total, total DEGs.

**Figure 3:**
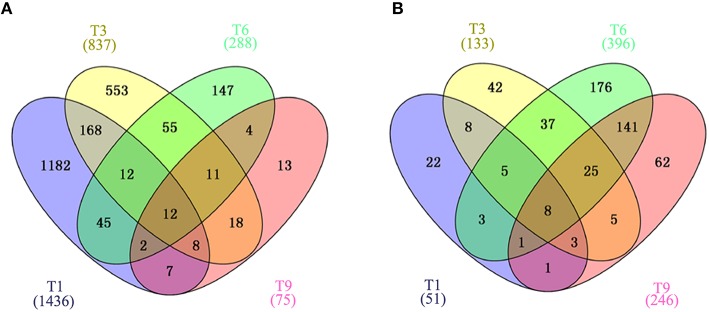
**Venn diagram showing the overlap of DEGs clustered into four comparison groups (T1, T3, T6, and T9) represented by four ellipses**. Numbers outside the ecllipses correspond to groups unique to a given infection stage. Numbers in the overlapping parts of different ellipses correspond to the number of DEGs in common from those comparison groups. **(A)** Veen diagram of identified DEGs in *T. gondii*. **(B)** Veen diagram of identified DEGs in PK-15 cells. Numbers in parentheses indicate the total number of DEGs in each data set.

Gene expression is calculated as FPKM and showed a comparable median distribution across different sequencing libraries (Figure S2). To characterize the expression patterns of mRNA activated during *T. gondii* infection, heat maps were constructed using FPKM values to determine overall transcriptome difference among different infection times PI. More than 80% of *T. gondii* genes were found to be expressed (FPKM>1). Heat map representation of differently expressed transcripts revealed a similarity in the trancriptomes within the early (T1, T3), the middle (T6, T9, T12) and the late (T18, T24) stages PI, respectively (Figure 2A). Parasites at stage T0 are just egressed from the host cells, which explains the clustering of the trascriptome at stage T0 with T18 and T24. Meanwhile, >50% of the PK-15 cell genes in the genome were found to be expressed (FPKM>1). Heat map based on the log_2_ FPKM values of differently expressed transcripts in PK-15 cells showed a similar cluster profile, except PK-15 cell transcriptome at T0, T1, and T3, which clustered together (Figure 2B). For differential gene expression analysis, pair wise comparisons of datasets from *T. gondii* during cell infection vs. egressed parasites (T0-T) and infected host cells vs. controls (T0-C) were performed, respectively. As shown in Figure 2C, more than half of the parasite's DEGs are down-regulated and the number of DEGs decreased in a time-dependent manner. However, the number of identified host DEGs fluctuated and peaked at T6, which was near the end of the first parasite cell cycle (Figure 2D). We further compared the DEGs in the parasites and host cells during the first *T. gondii* cell cycle (T1~T9), and the numbers of DEGs among these samples are visualized in the Venn diagrams. As shown in Figure 3A, DEGs belonging to a specific infection stage decrease in a time-dependent manner and there are 12 *T. gondii* DEGs that varied at all four infection stages. Meanwhile, 8 host DEGs varied during the process (Figure 3B). The validity of the differential expression of these DEGs that varied during the first four time points PI was confirmed by qRT-PCR analysis. The results of the qRT-PCR analysis agreed with the Illumina sequencing data, which are shown in Tables S2, S3.

### Gene ontology enrichment analysis

**Figure 4:**
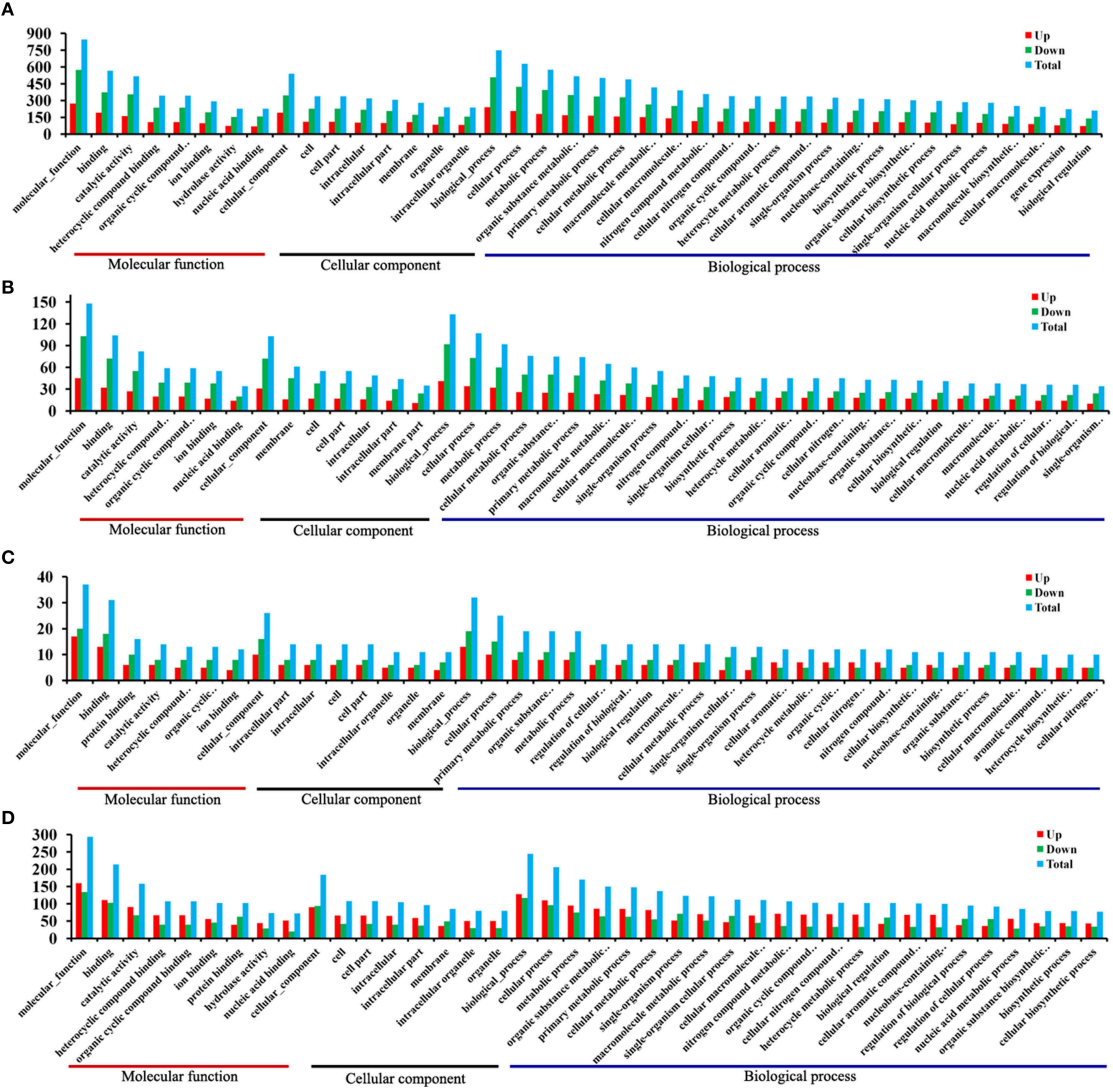
**DEGs GO cluster distribution**. DEGs were classified into three main categories: biological process, cellular component and molecular function. Shown are the identified functions and the corresponding numbers of DEGs for each GO category. **(A)** T1/T0-T DEGs GO cluster distribution. **(B)** T6/T0-T DEGs GO cluster distribution. **(C)** T1/T0-C DEGs GO cluster distribution. **(D)** T6/T0-C DEGs GO cluster distribution. The *y*-axis indicates the number of genes in a specific category. X-axis indicates different GO terms. Top 40 enriched GO terms are listed.

A total of 1436 *T. gondii* genes were considered to be significant when we compared T1 to T0-T. Of these, 524 genes were up-regulated and 912 were down-regulated. Gene GO is an international standardized gene functional classification system, which was applied to search for significantly enriched GO terms in DEGs. To obtain a comprehensive view of DEGs, we carried out the GO analysis using GOseq R package. Go term enrichment analysis was carried out to evaluate significantly over-represented GO terms of these 1436 DEGs and has resulted in a total of 2169 GO terms, including 1194 biological process terms, 304 cellular component terms and 671 molecular function terms. Each DEG could be assigned to more than one GO terms and the number of enriched GO terms was higher for down-regulated genes than for up-regulated genes. The top 40 enriched GO terms are shown in Figure 4A. The majority of the DEGs were classified under the “molecular function”. Within the three main categories of the GO classification, the “cellular process,” “cell,” and “binding” terms were most prevalent. We also noted that a high percentage of DEGs were classified under “metabolic process” and “catalytic activity,” while only a few DEGs were classified under “gene expression” and “biological regulation.” As infection progresses the number of DEGs decrease. A total of 288 DEGs were identified between T6 and T0-T, and these genes were categorized into 1100 GO terms. “Binding”, “membrane” and “cellular process” were the highest enrichment GO terms in the three main categories (Figure 4B).

Meanwhile, a total of 51 DEGs in the host cells were found between group T1 and T0-C. These genes were annotated to 522 GO terms, and “binding,” “intracellular” and “cellular processes” were the highest enrichment terms in the three main categories (Figure 4C). We then noted that in the host PK-15 cells the total number of DEGs between T6 and T0-C was 396 (205 up-regulated vs. 191 down-regulated). Go term enrichment analysis resulted in a total of 1491 GO terms, including 792 biological process terms, 240 cellular component terms and 459 molecular function terms. In contrast with the comparison between T1 and T0-C, the number of enriched GO terms in T6/T0-C was lower for down-regulated genes than for up-regulated genes, and the majority of the DEGs were classified under “molecular function,” especially under “binding” and “catalytic activity” (Figure 4D).

### Kyoto encyclopedia of genes and genomes (KEGG) pathway analysis

**Figure 5:**
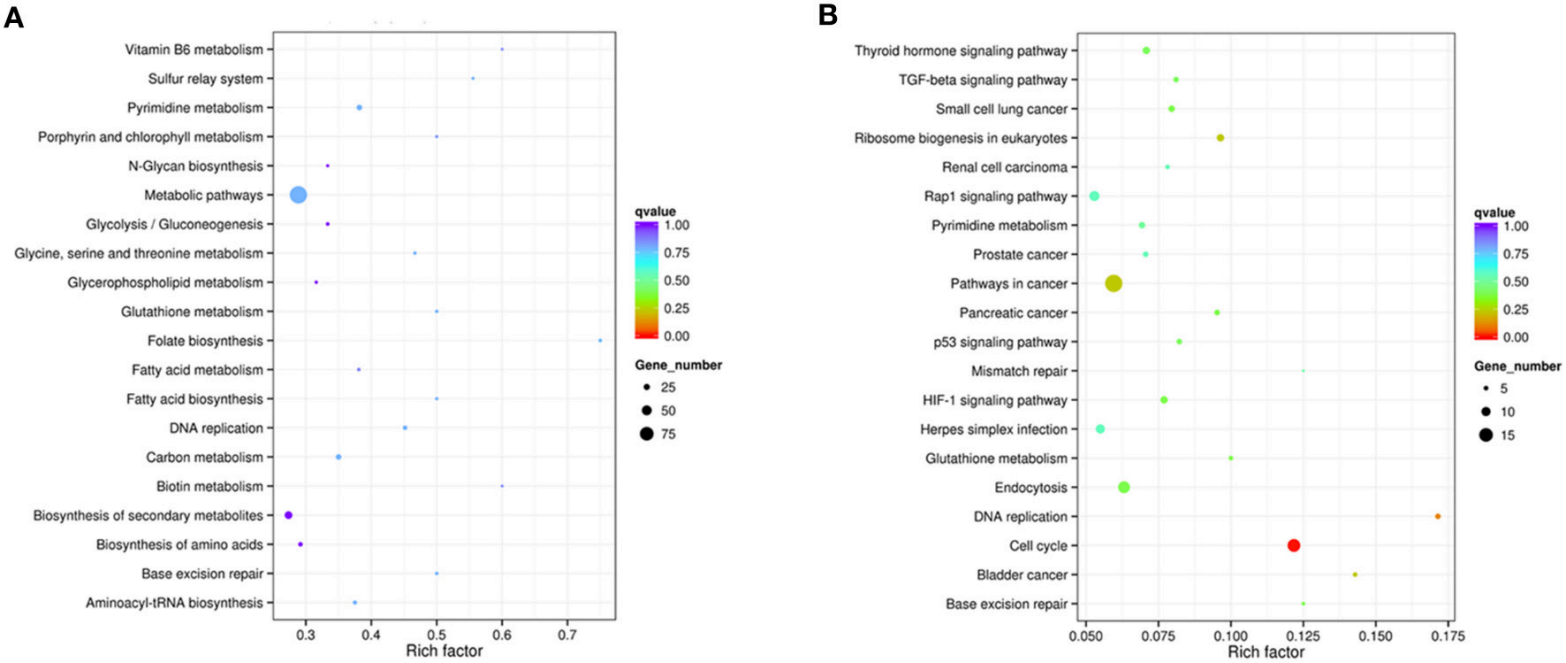
**Statistics of KEGG pathway enrichment**. The x-axis shows the enrichment factor. The y-axis corresponds to KEGG Pathway. The color of the dot represents q value and size of the dot represents the number of DEGs mapped to the reference pathways. **(A)** Top 20 enriched pathways in *T. gondii* at T1. **(B)** Top 20 enriched pathways in PK-15 cell at T6.

**Figure 6:**
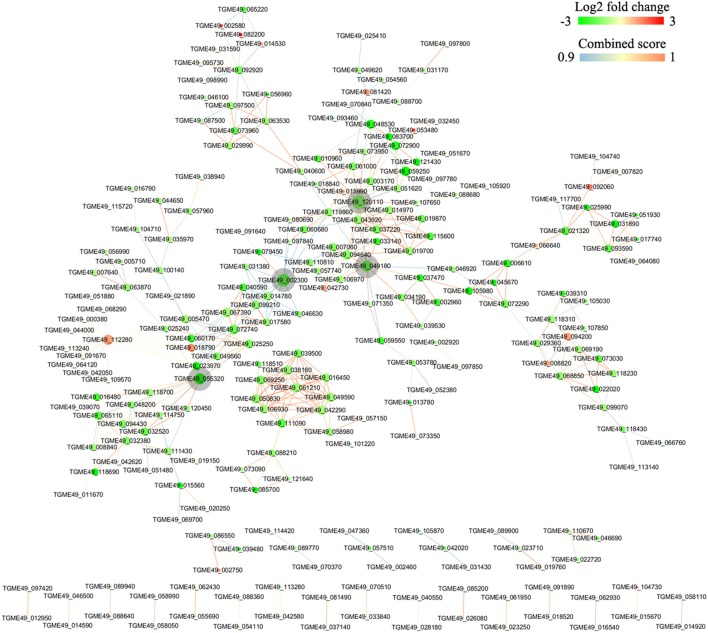
**Protein-protein interaction (PPI) networks of the DEGs identified in the comparison between T1 and T0-T**. The PPI network with a high combined score >0.9 was prepared using the STRING 10 database program. DEGs are represented as round nodes. The red node indicates upregulated or green node indicates downregulated EDGs. The node size indicates high interaction degree (large) or low degree (small). Proteins that are associated to each other are linked by an edge. The color of the edge indicates the combined interaction score.

DEGs were mapped to the reference pathway in the KEGG database in order to identify the parasite biological pathway operating during infection process. KEGG pathways analysis of *T. gondii* DEGs showed that only small number of DEGs was annotated to the KEGG pathways and most of which were related to metabolism. During the intracellular infection cycle of *T. gondii* a total of 41 metabolic pathways were predicted (Table S4). The “pyrimidine metabolism,” “carbon metabolism,” “biosynthesis of secondary metabolites,” “purine metabolism,” and “biosynthesis of amino acids” were highly enriched. Of the 1436 DEGs between T1 and T0-T, 203 genes had a KO ID and were associated with 68 pathways (23 up-regulated genes categorized into 24 pathways, and 180 down-regulated genes categorized into 68 pathways). The top 20 enriched pathways were shown in Figure 5A and all the enriched pathway were closely related to metabolic activities except “sulfur relay system” and “base excision repair.” Additionally, “metabolic pathway” included 99 DEGs, account for half of those genes with KO ID. The cytoscape software was used to visualize the protein-protein interaction networks of the products of DEGs. As shown in Figure 6, *TGME49_120110* (*PCNA*), *TGME49_049180* (*DHFR-TS*), *TGME49_055320* (hypothetical protein), and *TGME49_002300* (*ITPase*) are the four hubs with most interactions.

**Figure 7:**
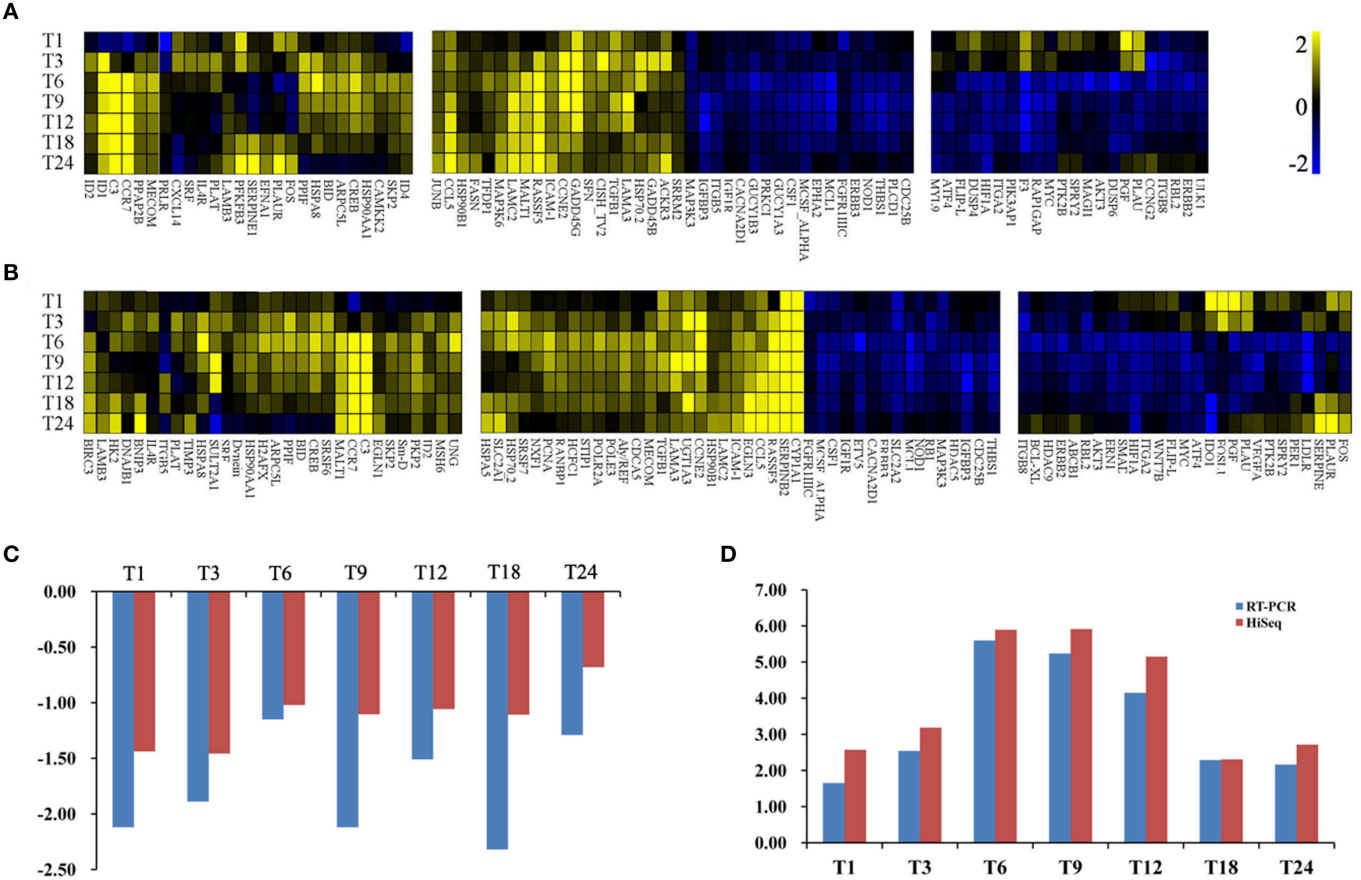
**Heat maps of the fold changes of immune (A) or disease (B) related genes with significantly changing expression levels across the entire infection course**. DEGs have been divided into three parts: the left part indicates genes that are upregulated at most infection times; the middle part indicates genes that are upregulated or downregulated at all infection times; the right part indicates genes that are downregulated at most infection times. The range of expression is represented by a color grade ranging from low (blue) to high (yellow). The x-axis indicates DEGs and y-axis means different samples. **(C)** Relative abundance of SLC2A2 as determined by RT-PCR and HiSeq analysis. X-axis indicates different samples. Y-axis indicates log_2_ fold change values. **(D)** Relative abundance of SERPINB2 as determined by RT-PCR and HiSeq analysis. X-axis indicates different samples. Y-axis indicates log_2_ fold change values.

To further investigate the biochemical pathways of DEGs in the host cells during *T. gondii* infection, all host cell DEGs were mapped to terms in the KEGG database and compared with the whole transcriptome background. In order to get a better understanding of the defense system in host cells during infection we identified DEGs and KEGG pathways related to immune response or disease activities. As shown in Table 2, a total of 27 enriched immune-relevant pathways from the KEGG database were predicted. There was large number of DEGs at the infection stage T6 and T9, especially at stage T6. Of the 396 DEGs between T6 and T0-C, 157 genes had a KO ID and were associated with 194 pathways (87 up-regulated genes involved in 142 pathways, and 70 down-regulated genes involved in 134 pathways). The top 20 enriched pathways are shown in Figure 5B, with DEGs involved in disease-related “small cell lung cancer,” “renal cell carcinoma,” “prostate cancer,” “pancreatic cancer,” and “bladder cancer” being highly enriched. Additionally, among theses DEGs, some immune-related molecules involved in “TGF-β signaling pathway,” “p53 signaling pathway,” and “rap1 signaling pathway” were identified through KEGG enrichment. Additionally, variations of log_2_ fold changes of 95 genes that are involved in immune-related pathways are present in Figure 7A. The majority of these genes are active throughout the entire infection course except at stage T1. Meanwhile, the alterations of 99 genes that are involved in disease-related pathways are shown in Figure 7B, which shows a similar profile. To confirm the differential gene expression from the transcriptome data, real-time PCR was performed to validate the expression of the selected *SLC2A2* and *SERPINB2*. As shown in Figures 7C,D, the expression patterns of these two DEGs were consistent with the high-throughput sequencing data, indicating that the transcriptome sequencing data were reliable.

**Table 2:** **Statistics of the number of host cell DEGs involved in immune related pathways**.

**Term**	**T1**	**T3**	**T6**	**T9**	**T12**	**T18**	**T24**	**Background**
Adipocytokine signaling pathway	0	1	3	0	0	1	1	70
Antigen processing and presentation	0	2	3	2	0	0	0	68
Apoptosis	0	0	4	1	1	1	1	91
B cell receptor signaling pathway	2	1	2	0	1	0	1	72
cAMP signaling pathway	2	1	3	1	1	0	2	201
Chemokine signaling pathway	1	2	2	2	2	2	2	173
Complement and coagulation cascades	2	3	3	4	4	4	4	73
Cytokine-cytokine receptor interaction	2	4	3	6	4	2	3	223
Cytosolic DNA-sensing pathway	0	0	0	1	1	1	1	61
Fc epsilon RI signaling pathway	1	1	1	0	0	0		63
Fc gamma R-mediated phagocytosis	1	1	3	1	0	0	0	81
Intestinal immune network for IgA production	1	1	0	1	1	0	0	44
Jak-STAT signaling pathway	1	2	4	1	0	0	0	153
MAPK signaling pathway	4	6	8	7	3	2	2	249
mTOR signaling pathway	0	0	4	2	2	1	1	60
Natural killer cell mediated cytotoxicity	1	1	3	0	0	0	1	108
NF-kappa B signaling pathway	1	1	4	2	1	1	2	90
NOD-like receptor signaling pathway	0	0	2	5	3	2	3	50
p53 signaling pathway	0	5	6	5	3	2	1	73
PI3K-Akt signaling pathway	3	3	15	13	8	0	4	328
Platelet activation	0	0	5	4	2	0	0	132
Rap1 signaling pathway	2	1	11	7	4	2	3	208
Ras signaling pathway	2	0	8	4	2	1	2	223
T cell receptor signaling pathway	2	1	3	1	0	0	1	107
TGF-beta signaling pathway	2	3	6	4	3	1	1	74
TNF signaling pathway	1	0	6	5	2	2	5	109
Toll-like receptor signaling pathway	1	0	1	1	1	1	2	101

## Discussion

Despite being recognized as the most common source of food-borne toxoplasmosis, very little is known about the mechanisms of *T. gondii* infection in pigs. Transcriptomic studies can provide useful information on the underlying pathogenic mechanisms and interactions following the course of *T. gondii* infection. Indeed, gene expression patterns of *T. gondii* and host cells have already been investigated (He et al., 2016; Zhou et al., 2016). However, apart from one study that has taken a combined host and parasite transcriptional profiling approach (Melo et al., 2013), previous studies have focused on either the host cells or the parasite, which has largely caused knowledge gap in the host-pathogen interactome. To address this need and to elucidate the temporally dynamic gene expression patterns of both host cells and *T. gondii*, transcriptome sequencing and differential gene expression analysis of *T. gondii* infecting swine PK-15 cells were performed through the course of an *in vitro* infection.

Our combined transcriptome analysis has identified distinct mRNA profiles in host PK-15 cells in response to *T. gondii* infection and has elucidated the molecular pathways and gene transcripts involved in innate defenses of PK-15 cells infected with *T. gondii*. Parasite infection induced substantial changes in the host cell transcriptome and revealed time-specific transcripts. Despite continuous infection for 24 h, the abundance of gene transcripts exhibited a dramatic change within the first hour PI, which decreased as infection time increased (Figure 2). These findings are consistent with a previous study, which also reported a time-dependent reprogramming of host transcriptomes in response to *T. gondii* infection (Zhou et al., 2016).

To determine the DEGs profile of *T. gondii*, cDNA were prepared from samples collected at different time points PI and sequenced using Illumina technology. Hierarchical clustering to group genes according to their level of expression (log-transformed FPKM values) provided an overall view of the parasite transcriptomic landscape. This analysis revealed that early infection is associated with the most DEGs, which are more likely to play a role in cell invasion and PV formation. Surprisingly, early infection (T1/T0-C) did not induce significant changes in gene expression of host cells, indicating that *T. gondii* invasion occurs in a subtle manner to maneuver the host defense surveillance. However, host cell DEGs peaked at stage T6, a period near the end of the first parasite cell cycle.

The KEGG pathway-based analysis was performed to better understand the DEGs functions and interactions. Among the 68 KEGG pathways to which obtained DEGs between group T1 and T0-T were assigned, “metabolic pathways” represented the largest category, followed by “biosynthesis of secondary metabolites,” “pyrimidine metabolism,” and “carbon metabolism.” Of the 68 KEGG pathways, 41 metabolic pathways were identified, implying that *T. gondii* tachtzoites can affect the host by altering the host cell microenvironment for their own benefit. PPI analysis revealed that *DHFR-TS* is a hub gene, which plays an important role in parasite invasion, and it is recognized as a crucial drug target due to its involvement in the folate metabolism and nucleotide synthesis (Sharma et al., 2013). Another hub gene, *PCNA*, codes for an evolutionarily conserved protein in all eukaryotic species and plays key roles in DNA replication, chromatin remodeling, DNA repair, and cell cycle regulation (Strzalka and Ziemienowicz, 2011).

The activation of anti-*T. gondii* immune response requires precise regulation of host cellular mRNAs expression, and any aberrations in the host cellular mRNAs expression during *T. gondii* infection may impair fine mechanism controlling host anti-*T. gondii* immunity. Upon entry into host cell, *T. gondii* did not induce significant DEGs in host cells until stage T6. The DEGs identified were involved in essential biological pathways and differential expression analysis showed host immune genes, such as *JUNB, CCL5, HSP90B1, FASN, TFDP1, MAP3K6, LAMC2, MALT1, RASSF5, ICAM-1, CCNE2, GADD45G, SFN, CISH_TV2, TGFB1, LAMA3, HSP70.2, GADD45B, ACKR3*, and *SRRM2* to be up-regulated throughout the infection cycle. In contrast, genes like *MAP3K3, IGFBP3, ITGB5, IGF1R, CACNA2D1, GUCY1B3, PRKC1, GUCY1A3, CSF1, MCSF_ALPHA, EPHA1, MCL1, FGFR1IIC, ERBB3, NOD1, THBS1, PLCD1*, and *CDC25B* were down-regulated.

We further analyzed the alterations of disease related genes in PK-15 cells. *CYP1A1* and *SERPINB2* displayed high expression levels along with *T. gondii* infection, and these two genes are involved in chemical carcinogenesis and amoebiasis, respectively (Faust and Guillen, 2012; Modi et al., 2012). Also, *SLC2A2* showed decreased expression levels; the product of this gene plays an important role in glycogen storage disease (Santer et al., 2002). These results further substantiate the dynamic host-pathogen cross-talks that occur during *T. gondii* infection.

In summary, the present study provided the first combined RNA-seq-based transcriptomic analysis of *T. gondii* and host cells during the first 24 h of infection and the first in-depth analysis of the molecular mechanisms *T. gondii* uses to establish itself within porcine kidney cells. *T. gondii* infection induced significant changes in porcine PK-15 cell's transcriptome concurrent with considerable reprogramming of metabolic processes and signaling regulatory pathways in *T. gondii*. Immune- and disease-related DEGs in PK-15 cells were induced during the entire infection course (24 h), except at the beginning of infection, supporting the crucial role played by the host immune defenses against *T. gondii* infection. Parasite DEGs involved in metabolic pathways showed significant changes at the onset of the infection. These findings indicate the gene expression of PK-15 cells engaging with *T. gondii* is tightly linked. Future studies should employ a systems biology functional analysis pipeline to characterize not only the functions of the identified genes, but also the linked proteins and metabolites during *T. gondii*-host interactions. These would in turn shed light on key processes that underpin the inter-kingdom signaling communication between this highly zoonotic apicomplexan parasite and the mammalian host. Ultimately, this may lead to identification of new diagnostic biomarkers of *T. gondii* infection or novel therapeutic targets.

## Author contributions

XZ, XS conceived and designed the experiments. CZ, QL performed the experiments. CZ analyzed the data. DZ contributed reagents/materials/analysis tools. CZ, HE wrote the paper. XZ contributed to the revision. All authors read and approved the final manuscript.

### Data availability statement

The data sets supporting the results of this article are available in the NCBI SRA repository with the accession number SRP063957.

### Conflict of interest statement

The authors declare that the research was conducted in the absence of any commercial or financial relationships that could be construed as a potential conflict of interest.

## Supplementary material

Figure S1 **Percent of reads uniquely mapped to reference genomes compared to total mapped**. Tg reads that were mapped to *T. gondii* genome; C, reads that were mapped to pig genome. X axis, time post infection (h); y axis, percent of mapped reads.

Figure S2 **The comparisons of gene expression levels among samples examined at different time points post infection (PI) and controls based on FPKM values. (A)** Box plots compare the levels of actively expressed genes of parasites at different time points PI. **(B)** Box plots compare the levels of actively expressed genes of host PK-15 cells at different time points PI. X axis indicates different samples; y axis indicates log_10_ (FPKM+1) values in RNA-Seq libraries. T0-T indicates reads library from *T. gondii* control sample. T0-C indicates read library from PK-15 cell control sample.

Table S1 **List of primers used in real-time quantitative RT-PCR analysis**.

Table S2 **Real-time qRT-PCR of DEGs in the parasites that vary at all four infection stages (T1, T3, T6, and T9)**.

Table S3 **Real-time qRT-PCR of DEGs in the host PK-15 cells that vary at all four infection stages (T1, T3, T6, and T9)**.

Table S4 **Statistics of the number of parasite DEGs involved in metabolic activities**.

## Abbreviations

ACKR3: atypical chemokine receptor 3
CACNA2D1: calcium voltage-gated channel auxiliary subunit alpha 2 delta 1
CCL5: C-C motif chemokine ligand 5
CCNE2: cyclin E2
CDC25B: cell division cycle 25B
CISH_TV2: cytokine inducible SH2-containing protein
CSF1: colony stimulating factor 1
CYP1A1: cytochrome P450 family 1 subfamily A member 1
DHFR-TS: bifunctional dihydrofolate reductase-thymidylate synthase
EPHA1: EPH receptor A1
ERBB3: erb-b2 receptor tyrosine kinase 3
FASN: fatty acid synthase
FGFR1IIC: fibroblast growth factor receptor 1
GADD45B: growth arrest and DNA damage inducible beta
GADD45G: growth arrest and DNA damage inducible gamma
GUCY1A3, guanylate cyclase 1, soluble: alpha 3
GUCY1B3, guanylate cyclase 1, soluble: beta 3
HSP70.2: heat shock 70 kDa protein 1B
HSP90B1: heat shock protein 90kDa beta family member 1
ICAM-1: intercellular adhesion molecule 1
IGFBP3: insulin like growth factor binding protein 3
IGF1R: insulin like growth factor 1 receptor
ITGB5: integrin subunit beta 5
ITPase: inosine triphosphate pyrophosphatase
JUNB: jun B proto-oncogene
LAMA3: laminin subunit alpha 3
LAMC2: laminin subunit gamma 2
MALT1: mucosa-associated lymphoid tissue lymphoma translocation protein 1
MAP3K3: mitogen-activated protein kinase kinase kinase 3
MAP3K6: mitogen-activated protein kinase kinase kinase 6
MCL1: myeloid cell leukemia 1
MCSF_ALPHA: macrophage colony stimulating factor alpha
NOD1: nucleotide binding oligomerization domain containing 1
PCNA: proliferating cell nuclear antigen
PLCD1: phospholipase C delta 1
PRKC1: protein kinase C1
RASSF5: Ras association domain family member 5
SERPINB2: serpin family B member 2
SFN: stratifin
SLC2A2: solute carrier family 2 member 2
SRRM2: serine/arginine repetitive matrix 2
TFDP1: transcription factor Dp-1
TGFB1, transforming growth factor beta 1 and THBS1: thrombospondin 1.
