# Supplementary figures and images for: Dual Identification and Analysis of Differentially Expressed Transcripts of Porcine PK-15 Cells and Toxoplasma gondii during in vitro Infection
Source: Front Microbiol. 2016 May 13;7:721. doi: 10.3389/fmicb.2016.00721 (PMC4865485; doi:10.3389/fmicb.2016.00721)

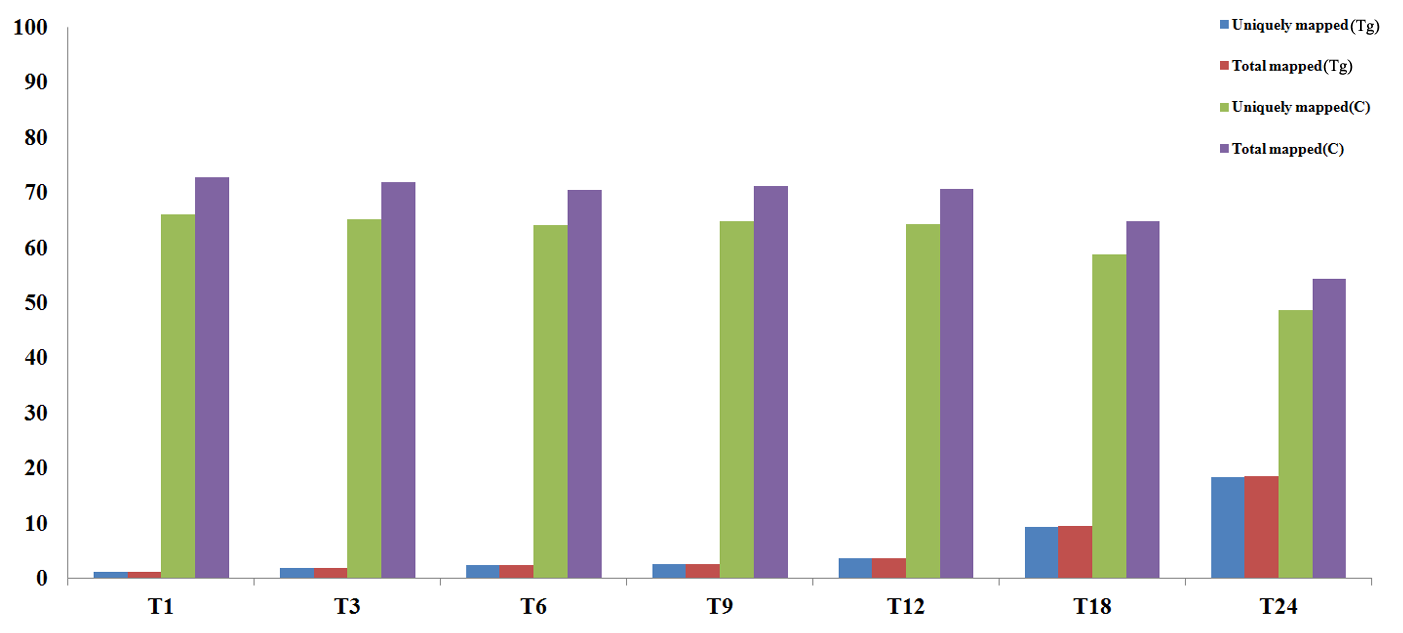

Supplement: Figure S1 — Percent of reads uniquely mapped to reference genomes compared to total mapped. Tg reads that were mapped to T. gondii genome; C, reads that were mapped to pig genome. X axis, time post infection (h); y axis, percent of mapped reads. [file Image1.TIF]

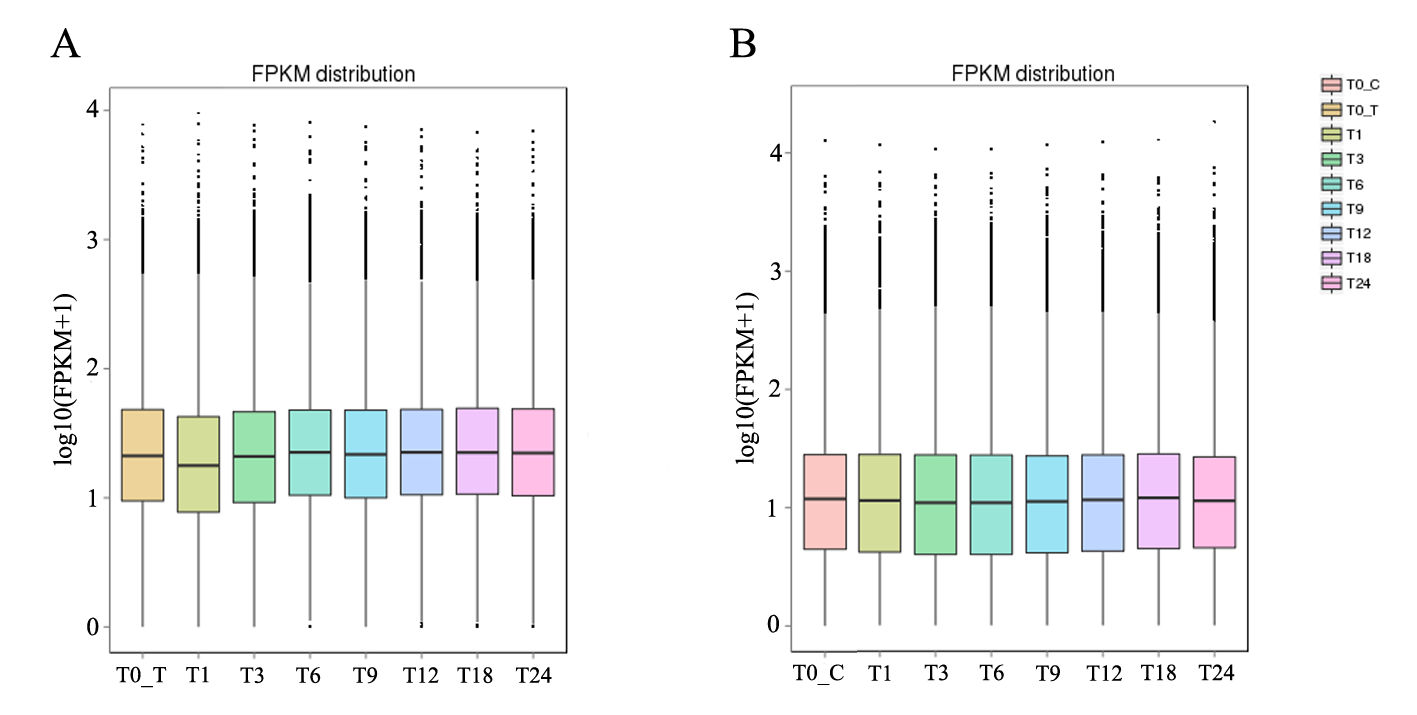

Supplement: Figure S2 — The comparisons of gene expression levels among samples examined at different time points post infection (PI) and controls based on FPKM values. (A) Box plots compare the levels of actively expressed genes of parasites at different time points PI. (B) Box plots compare the levels of actively expressed genes of host PK-15 cells at different time points PI. X axis indicates different samples; y axis indicates log10 (FPKM+1) values in RNA-Seq libraries. T0-T indicates reads library from T. gondii control sample. T0-C indicates read library from PK-15 cell control sample. [file Image2.TIF]
