# Supplementary material for: Dual Identification and Analysis of Differentially Expressed Transcripts of Porcine PK-15 Cells and Toxoplasma gondii during in vitro Infection
Source: Front Microbiol. 2016 May 13;7:721. doi: 10.3389/fmicb.2016.00721 (PMC4865485; doi:10.3389/fmicb.2016.00721)
Supplement: Table S1 — List of primers used in real-time quantitative RT-PCR analysis. [file Table1.DOC]

**TableS s1｜List of primers used in real-time quantitative RT-PCR analysis.**

| **GeneBank Accession** | **Primers** | **Sequences** |
| --- | --- | --- |
| XM_013990435.1 | ADAMTS14-F | 5’-GACACTTTGAATGATGGTTGTGGT -3’ |
|  | ADAMTS14-R | 5’-TGCTGTCTGTGCGGATGAG -3’ |
| NM_213922.1 | ANKRD1-F | 5’-GGATGATTGTTGCCCTCTTTTC -3’ |
|  | ANKRD1-R | 5’- AATGGTTTGCTTGTCCTTCTCAC-3’ |
| XM_005669358.2 | CTDSPL-F | 5’-CAGTTCTTTCTTCTGCTGCTTCC -3’ |
|  | CTDSPL-R | 5’- CCACCATTCTCCTCCACCA-3’ |
| XM_001927740.4 | CYR61-F | 5’-TCTCTACACCCCTCCTCTGCTC -3’ |
|  | CYR61-R | 5’- GCTGCATCTCTTGCCCTTTT-3’ |
| XM_013977473.1 | DCDC2-F | 5’-GAACTCACCACCAACAGTTCCA -3’ |
|  | DCDC2-R | 5’-CTCTCCATCTTCTTCCTCATCCA -3’ |
| XM_013979006.1 | DDIT4L-F | 5’-GTAGTTCACCCTGCCTGCTCTT -3’ |
|  | DDIT4L-R | 5’- CTTTTCCTTCCTTATTCCATTCCAC-3’ |
| NM_213785.1 | F3-F | 5’-GACCATTTTGGAGTGGGAACC -3’ |
|  | F3-R | 5’- CCGTGTCTGTCGTGTGGAA-3’ |
| XM_003121697.3 | SERPINB2-F | 5’-CCTGTATCTGTCCCAAGTGTTCC -3’ |
|  | SERPINB2-R | 5’-GGAAGAGGAAGGGGTGGTCT -3’ |
| EF140874.1 | SLC2A2-F | 5’-CTGAGGAAGAGACTACGGCATC -3’ |
|  | SLC2A2-R | 5’-CTCCAAGCATCCCACCAAA -3’ |
| XM_013991900.1 | UPP1-F | 5’-TTCTGCCTGGGCTGTGTTT -3’ |
|  | UPP1-R | 5’-TGTTTCTGGGGTGTGGTTTG -3’ |
| NM_001206359.1 | GAPDH (PK15) -F | 5’-TGGAGTCCACTGGTGTCTTCAC |
|  | GAPDH (PK15) -R | 5’-TTCACGCCCATCACAAACA |
| XM_002369602.1 | TG008730-F | 5’-TCGGCGACGACAATAGTAACAG |
|  | TG008730-R | 5’-CGGTGCGAGTTGGGATTT |
| XM_002369706.1 | TG009980-F | 5’-TTTCCCTGTTTGGTCGTTCC |
|  | TG009980-R | 5’-GAATGCGGCGTCAAAATCTC |
| XM_002365980.1 | TG023250-F | 5’-GTGGATTCGCTTGTATTTCTTCTTC |
|  | TG023250-R | 5’-GGACACATCTTTGGCTTGTAGG |
| XM_002366266.1 | TG026540-F | 5’-TCTGATTCGGTCGATGATTGTAG |
|  | TG026540-R | 5’-CGATGCTGTTGTAGGCGATTT |
| XM_002367865.1 | TG029720-F | 5’-AAATGGAGGCAGAGAGATGAGTG -3’ |
|  | TG029720-R | 5’- AGCGAAAAGCAGCAAAGAGG-3’ |
| XM_002366741.1 | TG042620-F | 5’-CAGCAGTCCAAGCATCGTG -3’ |
|  | TG042620-R | 5’-TGCCTCGTTCTCCAGTCGT -3’ |
| XM_002365807.1 | TG072040-F | 5’-CAGCGAAGGAGCAAAAGAAAC |
|  | TG072040-R | 5’-GCGAGACCAAAAGCCAAATC |
| XM_002370115.1 | TG093540-F | 5’-CAACAGGAAAAGGCGAGAATG |
|  | TG093540-R | 5’-GTCAGTTCAGGAGGAAGGGAGA |
| XM_002370383.1 | TG106620-F | 5’-TCCTGGCGGGAAAGAAAA |
|  | TG106620-R | 5’-GAAGAACGGGACGGCAAA |
| XM_002371629.1 | TG101580-F | 5’-AAAAGAGACCGGCCAGACAA -3’ |
|  | TG101580-R | 5’-CGAAGGAGAAGGACAACAGAGAA -3’ |
| XM_002364279.1 | TG110740-F | 5’-CGACAAGAGGCGACCAGAA |
|  | TG110740-R | 5’-AAAGCCCACGCAACAGAGA |
| XM_002364339.1 | TG111380-F | 5’-CCTTCGTGTTTGCCTCTACAAAA |
|  | TG111380-R | 5’-GCTGAAAATGCTACCCCAACTC |
| AF265361.1 | GAPDH (*T. gondii*) -F | 5’-GCGAAGTCAGCCACAAGGA -3’ |
|  | GAPDH (*T. gondii*) -R | 5’-CGGTGGACTCGCAGATGTAG -3’ |
