# Supplementary material for: Dual Identification and Analysis of Differentially Expressed Transcripts of Porcine PK-15 Cells and Toxoplasma gondii during in vitro Infection
Source: Front Microbiol. 2016 May 13;7:721. doi: 10.3389/fmicb.2016.00721 (PMC4865485; doi:10.3389/fmicb.2016.00721)
Supplement: Table S2 — Real-time qRT-PCR of DEGs in the parasites that vary at all four infection stages (T1, T3, T6, and T9). [file Table2.DOC]

**Table S2｜** Real-time qRT-PCR of DEGs in the parasites that vary at all four infection stages (T1, T3, T6 and T9).

| **Genes** | **GeneBank Accession** | **T1^a^** | **T1^b^** | **T3^a^** | **T3^b^** | **T6^a^** | **T6^b^** | **T9^a^** | **T9^b^** |
| --- | --- | --- | --- | --- | --- | --- | --- | --- | --- |
| TG008730 | XM_002369602.1 | 4.74 | 3.35 | 5.16 | 2.62 | 4.45 | 1.66 | 4.17 | 1.79 |
| TG009980 | XM_002369706.1 | 3.57 | 3.00 | 4.53 | 2.46 | 4.08 | 2.71 | 3.83 | 2.65 |
| TG023250 | XM_002365980.1 | 0.02 | 0.08 | -4.14 | -5.44 | -4.73 | -5.48 | -2.67 | -3.74 |
| TG026540 | XM_002366266.1 | 1.00 | 1.03 | -1.87 | -1.78 | -1.57 | -1.47 | -0.82 | -1.11 |
| TG029720 | XM_002367865.1 | 3.17 | 1.47 | 2.80 | 1.47 | 3.36 | 2.09 | 2.65 | 1.47 |
| TG042620 | XM_002366741.1 | 2.24 | 1.10 | 1.86 | 2.01 | 0.35 | 1.26 | 0.85 | 1.16 |
| TG072040 | XM_002365807.1 | 0.26 | 1.44 | 0.65 | 1.41 | 0.66 | 1.62 | 0.51 | 1.62 |
| TG093540 | XM_002370115.1 | -0.01 | -1.52 | -1.58 | -1.35 | -1.56 | -1.50 | -1.49 | -2.14 |
| TG106620 | XM_002370383.1 | -0.64 | -1.99 | 1.55 | 1.71 | 0.41 | 1.71 | 1.59 | 1.60 |
| TG101580 | XM_002371629.1 | 3.95 | 3.92 | 2.01 | 1.18 | 0.68 | 1.02 | 1.82 | 1.22 |
| TG110740 | XM_002364279.1 | -0.06 | -1.11 | -0.89 | -2.17 | -1.19 | -1.31 | -0.76 | -1.16 |
| TG111380 | XM_002364339.1 | 2.48 | 1.84 | 2.45 | 2.17 | 2.06 | 2.07 | 2.01 | 2.77 |

a qRT-PCR log_2_ fold-change. qRT-PCR results were determined from the log_2_ of the2^− ΔΔ CT^ values from the *T. gondii* infected samples divided by that from the controls

b HiSeq log_2_ fold-change. HiSeq log_2_ fold-change was determined by the log_2_ of the RPKM value from the *T. gondii*-infected samples divided by that of the controls.
