# Supplementary material for: Dual Identification and Analysis of Differentially Expressed Transcripts of Porcine PK-15 Cells and Toxoplasma gondii during in vitro Infection
Source: Front Microbiol. 2016 May 13;7:721. doi: 10.3389/fmicb.2016.00721 (PMC4865485; doi:10.3389/fmicb.2016.00721)
Supplement: Table S3 — Real-time qRT-PCR of DEGs in the host PK-15 cells that vary at all four infection stages (T1, T3, T6, and T9). [file Table3.DOC]

**Table S3｜ Real-time qRT-PCR of DEGs in the host PK-15 cells that vary at all four infection stages (T1, T3, T6 and T9).**

| **Genes** | **GeneBank Accession** | **T1^a^** | **T1^b^** | **T3^a^** | **T3^b^** | **T6^a^** | **T6^b^** | **T9^a^** | **T9^b^** |
| --- | --- | --- | --- | --- | --- | --- | --- | --- | --- |
| ADAMTS14 | XM_013990435.1 | -0.39 | -1.00 | -0.06 | -1.38 | -1.72 | -0.69 | -0.94 | -1.82 |
| ANKRD1 | NM_213922.1 | -2.49 | -2.70 | -3.30 | -3.04 | -2.34 | -2.64 | -3.18 | -2.92 |
| CTDSPL | XM_005669358.2 | -1.63 | -1.44 | -1.27 | -1.15 | -1.07 | -0.92 | -1.76 | -1.20 |
| CYR61 | XM_001927740.4 | -0.83 | -1.38 | -2.28 | -1.62 | -2.78 | -3.32 | -4.25 | -3.23 |
| DCDC2 | XM_013977473.1 | -0.30 | -1.66 | -1.16 | -1.27 | -1.85 | -1.56 | -3.42 | -1.53 |
| DDIT4L | XM_013979006.1 | -0.98 | -1.22 | -1.98 | -1.99 | -1.32 | -1.29 | -2.80 | -1.45 |
| F3 | NM_213785.1 | 1.06 | 1.32 | 0.64 | 1.18 | -2.28 | -2.56 | -3.02 | -2.53 |
| UPP1 | XM_013991900.1 | 1.57 | 1.63 | 1.10 | 1.43 | 2.02 | 0.08 | 3.1 | 2.38 |

a qRT-PCR log_2_ fold-change. qRT-PCR results were determined from the log_2_ of the2^− ΔΔ CT^ values from the *T.gondii* infected samples divided by that from the controls

b HiSeq log_2_ fold-change. HiSeq log_2_ fold-change was determined by the log_2_ of the RPKM value from the *T. gondii*-infected samples divided by that of the controls.
