# Supplementary material for: Dual Identification and Analysis of Differentially Expressed Transcripts of Porcine PK-15 Cells and Toxoplasma gondii during in vitro Infection
Source: Front Microbiol. 2016 May 13;7:721. doi: 10.3389/fmicb.2016.00721 (PMC4865485; doi:10.3389/fmicb.2016.00721)
Supplement: Table S4 — Statistics of the number of parasite DEGs involved in metabolic activities. [file Table4.doc]

**TableS s4｜Statistics of the number of parasite DEGs involved in metabolism.**

| **Term** | **T1** | **T3** | **T6** | **T9** | **Background** |
| --- | --- | --- | --- | --- | --- |
| [2-Oxocarboxylic acid metabolism](../../../../F:%5CNH150544_Toxoplasma_gondii_results%5C11.DEG_KEGGEnrichment%5C11.3.DEG_KEGGPath%5CALL%5CT1vsT0_T%5Csrc%5Ctgo01210.html) | 3 | 1 | 0 | 0 | 10 |
| [Alanine, aspartate and glutamate metabolism](../../../../F:%5CNH150544_Toxoplasma_gondii_results%5C11.DEG_KEGGEnrichment%5C11.3.DEG_KEGGPath%5CALL%5CT1vsT0_T%5Csrc%5Ctgo00250.html) | 4 | 2 | 0 | 0 | 12 |
| [Amino sugar and nucleotide sugar metabolism](../../../../F:%5CNH150544_Toxoplasma_gondii_results%5C11.DEG_KEGGEnrichment%5C11.3.DEG_KEGGPath%5CALL%5CT1vsT0_T%5Csrc%5Ctgo00520.html) | 4 | 1 | 0 | 0 | 16 |
| [Arginine and proline metabolism](../../../../F:%5CNH150544_Toxoplasma_gondii_results%5C11.DEG_KEGGEnrichment%5C11.3.DEG_KEGGPath%5CALL%5CT1vsT0_T%5Csrc%5Ctgo00330.html) | 1 | 0 | 1 | 0 | 11 |
| [Biosynthesis of amino acids](../../../../F:%5CNH150544_Toxoplasma_gondii_results%5C11.DEG_KEGGEnrichment%5C11.3.DEG_KEGGPath%5CALL%5CT1vsT0_T%5Csrc%5Ctgo01230.html) | 14 | 2 | 2 | 1 | 48 |
| [Biosynthesis of secondary metabolites](../../../../F:%5CNH150544_Toxoplasma_gondii_results%5C11.DEG_KEGGEnrichment%5C11.3.DEG_KEGGPath%5CALL%5CT1vsT0_T%5CT1vsT0_T.html) | 35 | 5 | 2 | 1 | 128 |
| [Biosynthesis of unsaturated fatty acids](../../../../F:%5CNH150544_Toxoplasma_gondii_results%5C11.DEG_KEGGEnrichment%5C11.3.DEG_KEGGPath%5CALL%5CT1vsT0_T%5Csrc%5Ctgo01040.html) | 2 | 1 | 0 | 0 | 7 |
| [Biotin metabolism](../../../../F:%5CNH150544_Toxoplasma_gondii_results%5C11.DEG_KEGGEnrichment%5C11.3.DEG_KEGGPath%5CALL%5CT1vsT0_T%5Csrc%5Ctgo00780.html) | 3 | 2 | 0 | 0 | 5 |
| Carbon metabolism | 21 | 4 | 1 | 1 | 60 |
| [Citrate cycle (TCA cycle)](../../../../F:%5CNH150544_Toxoplasma_gondii_results%5C11.DEG_KEGGEnrichment%5C11.3.DEG_KEGGPath%5CALL%5CT1vsT0_T%5Csrc%5Ctgo00020.html) | 5 | 1 | 0 | 0 | 21 |
| [Cysteine and methionine metabolism](../../../../F:%5CNH150544_Toxoplasma_gondii_results%5C11.DEG_KEGGEnrichment%5C11.3.DEG_KEGGPath%5CALL%5CT1vsT0_T%5Csrc%5Ctgo00270.html) | 2 | 0 | 0 | 0 | 10 |
| [Fatty acid biosynthesis](../../../../F:%5CNH150544_Toxoplasma_gondii_results%5C11.DEG_KEGGEnrichment%5C11.3.DEG_KEGGPath%5CALL%5CT1vsT0_T%5Csrc%5Ctgo00061.html) | 6 | 2 | 0 | 0 | 12 |
| [Fatty acid degradation](../../../../F:%5CNH150544_Toxoplasma_gondii_results%5C11.DEG_KEGGEnrichment%5C11.3.DEG_KEGGPath%5CALL%5CT1vsT0_T%5Csrc%5Ctgo00071.html) | 1 | 1 | 0 | 0 | 12 |
| [Fatty acid elongation](../../../../F:%5CNH150544_Toxoplasma_gondii_results%5C11.DEG_KEGGEnrichment%5C11.3.DEG_KEGGPath%5CALL%5CT1vsT0_T%5Csrc%5Ctgo00062.html) | 2 | 1 | 0 | 0 | 5 |
| [Fatty acid metabolism](../../../../F:%5CNH150544_Toxoplasma_gondii_results%5C11.DEG_KEGGEnrichment%5C11.3.DEG_KEGGPath%5CALL%5CT1vsT0_T%5Csrc%5Ctgo01212.html) | 8 | 3 | 0 | 0 | 21 |
| Folate biosynthesis | 9 | 0 | 0 | 0 | 12 |
| [Fructose and mannose metabolism](../../../../F:%5CNH150544_Toxoplasma_gondii_results%5C11.DEG_KEGGEnrichment%5C11.3.DEG_KEGGPath%5CALL%5CT1vsT0_T%5Csrc%5Ctgo00051.html) | 2 | 0 | 1 | 1 | 12 |
| Glutathione metabolism | 7 | 2 | 0 | 0 | 14 |
| [Glycerolipid metabolism](../../../../F:%5CNH150544_Toxoplasma_gondii_results%5C11.DEG_KEGGEnrichment%5C11.3.DEG_KEGGPath%5CALL%5CT1vsT0_T%5Csrc%5Ctgo00561.html) | 2 | 0 | 0 | 0 | 7 |
| [Glycerophospholipid metabolism](../../../../F:%5CNH150544_Toxoplasma_gondii_results%5C11.DEG_KEGGEnrichment%5C11.3.DEG_KEGGPath%5CALL%5CT1vsT0_T%5Csrc%5Ctgo00564.html) | 6 | 0 | 0 | 0 | 19 |
| [Glycine, serine and threonine metabolism](../../../../F:%5CNH150544_Toxoplasma_gondii_results%5C11.DEG_KEGGEnrichment%5C11.3.DEG_KEGGPath%5CALL%5CT1vsT0_T%5Csrc%5Ctgo00260.html) | 7 | 2 | 0 | 0 | 15 |
| [Glycolysis / Gluconeogenesis](../../../../F:%5CNH150544_Toxoplasma_gondii_results%5C11.DEG_KEGGEnrichment%5C11.3.DEG_KEGGPath%5CALL%5CT1vsT0_T%5Csrc%5Ctgo00010.html) | 9 | 0 | 1 | 1 | 27 |
| [Glycosylphosphatidylinositol (GPI)-anchor biosynthesis](../../../../F:%5CNH150544_Toxoplasma_gondii_results%5C11.DEG_KEGGEnrichment%5C11.3.DEG_KEGGPath%5CALL%5CT1vsT0_T%5Csrc%5Ctgo00563.html) | 2 | 0 | 1 | 0 | 10 |
| [Glyoxylate and dicarboxylate metabolism](../../../../F:%5CNH150544_Toxoplasma_gondii_results%5C11.DEG_KEGGEnrichment%5C11.3.DEG_KEGGPath%5CALL%5CT1vsT0_T%5Csrc%5Ctgo00630.html) | 3 | 1 | 0 | 0 | 9 |
| [Lysine degradation](../../../../F:%5CNH150544_Toxoplasma_gondii_results%5C11.DEG_KEGGEnrichment%5C11.3.DEG_KEGGPath%5CALL%5CT1vsT0_T%5Csrc%5Ctgo00310.html) | 1 | 1 | 0 | 0 | 6 |
| Metabolic pathways | 99 | 28 | 7 | 3 | 343 |
| [N-Glycan biosynthesis](../../../../F:%5CNH150544_Toxoplasma_gondii_results%5C11.DEG_KEGGEnrichment%5C11.3.DEG_KEGGPath%5CALL%5CT1vsT0_T%5Csrc%5Ctgo00510.html) | 5 | 3 | 0 | 0 | 15 |
| [Nicotinate and nicotinamide metabolism](../../../../F:%5CNH150544_Toxoplasma_gondii_results%5C11.DEG_KEGGEnrichment%5C11.3.DEG_KEGGPath%5CALL%5CT1vsT0_T%5Csrc%5Ctgo00760.html) | 2 | 2 | 0 | 0 | 5 |
| [Oxidative phosphorylation](../../../../F:%5CNH150544_Toxoplasma_gondii_results%5C11.DEG_KEGGEnrichment%5C11.3.DEG_KEGGPath%5CALL%5CT1vsT0_T%5Csrc%5Ctgo00190.html) | 6 | 2 | 1 | 1 | 40 |
| [Pantothenate and CoA biosynthesis](../../../../F:%5CNH150544_Toxoplasma_gondii_results%5C11.DEG_KEGGEnrichment%5C11.3.DEG_KEGGPath%5CALL%5CT1vsT0_T%5Csrc%5Ctgo00770.html) | 1 | 0 | 0 | 0 | 6 |
| [Porphyrin and chlorophyll metabolism](../../../../F:%5CNH150544_Toxoplasma_gondii_results%5C11.DEG_KEGGEnrichment%5C11.3.DEG_KEGGPath%5CALL%5CT1vsT0_T%5Csrc%5Ctgo00860.html) | 5 | 1 | 0 | 0 | 10 |
| [Propanoate metabolism](../../../../F:%5CNH150544_Toxoplasma_gondii_results%5C11.DEG_KEGGEnrichment%5C11.3.DEG_KEGGPath%5CALL%5CT1vsT0_T%5Csrc%5Ctgo00640.html) | 3 | 1 | 0 | 0 | 8 |
| [Purine metabolism](../../../../F:%5CNH150544_Toxoplasma_gondii_results%5C11.DEG_KEGGEnrichment%5C11.3.DEG_KEGGPath%5CALL%5CT1vsT0_T%5Csrc%5Ctgo00230.html) | 18 | 8 | 1 | 1 | 71 |
| Pyrimidine metabolism | 21 | 7 | 0 | 1 | 55 |
| [Pyruvate metabolism](../../../../F:%5CNH150544_Toxoplasma_gondii_results%5C11.DEG_KEGGEnrichment%5C11.3.DEG_KEGGPath%5CALL%5CT1vsT0_T%5Csrc%5Ctgo00620.html) | 5 | 0 | 0 | 0 | 16 |
| [Starch and sucrose metabolism](../../../../F:%5CNH150544_Toxoplasma_gondii_results%5C11.DEG_KEGGEnrichment%5C11.3.DEG_KEGGPath%5CALL%5CT1vsT0_T%5Csrc%5Ctgo00500.html) | 2 | 0 | 0 | 0 | 10 |
| [Sulfur metabolism](../../../../F:%5CNH150544_Toxoplasma_gondii_results%5C11.DEG_KEGGEnrichment%5C11.3.DEG_KEGGPath%5CALL%5CT1vsT0_T%5Csrc%5Ctgo00920.html) | 1 | 0 | 0 | 0 | 9 |
| [Terpenoid backbone biosynthesis](../../../../F:%5CNH150544_Toxoplasma_gondii_results%5C11.DEG_KEGGEnrichment%5C11.3.DEG_KEGGPath%5CALL%5CT1vsT0_T%5Csrc%5Ctgo00900.html) | 3 | 0 | 0 | 0 | 10 |
| [Tryptophan metabolism](../../../../F:%5CNH150544_Toxoplasma_gondii_results%5C11.DEG_KEGGEnrichment%5C11.3.DEG_KEGGPath%5CALL%5CT1vsT0_T%5Csrc%5Ctgo00380.html) | 1 | 1 | 0 | 0 | 5 |
| [Valine, leucine and isoleucine degradation](../../../../F:%5CNH150544_Toxoplasma_gondii_results%5C11.DEG_KEGGEnrichment%5C11.3.DEG_KEGGPath%5CALL%5CT1vsT0_T%5Csrc%5Ctgo00280.html) | 4 | 2 | 1 | 0 | 16 |
| [Vitamin B6 metabolism](../../../../F:%5CNH150544_Toxoplasma_gondii_results%5C11.DEG_KEGGEnrichment%5C11.3.DEG_KEGGPath%5CALL%5CT1vsT0_T%5Csrc%5Ctgo00750.html) | 3 | 2 | 0 | 0 | 5 |
